# Supplementary material for: Effects of smoking on genome-wide DNA methylation profiles: A study of discordant and concordant monozygotic twin pairs
Source: eLife. 2023 Aug 10;12:e83286. doi: 10.7554/eLife.83286 (PMC10501767; doi:10.7554/eLife.83286)
Supplement: Supplementary file 1. [file elife-83286-supp1.docx]

**Questions on smoking that were asked at blood draw**

“Did you ever smoke?”

(1) no, I never smoked

(2) I’m a former smoker

2a How many years ago did you quit?

2b For how many years have you smoked?

2c How many cigarettes did you use to smoke per day?

(3) yes.

3a How many years have you smoked?

3b How many cigarettes do you smoke per day?

**Quality control of longitudinal smoking data**

Out of 9628 participants of the NTR biobank 1 project, smoking status at blood draw was consistent with longitudinal surveys for 97.1% of participants. The remaining 2.9% included the following cases: For 0.3% the status at blood draw has been adjusted based on checks against longitudinal surveys (e.g. the person reported to have never smoked at blood draw, while they reported smoking in longitudinal surveys). For 2%, blood status at blood draw was missing, and has been added from survey data. For 0.1%, smoking status was set to missing due to unresolvable inconsistencies (such participants are not included in our study because of missing smoking status). For 0.4%, the status at blood draw was missing and it could also not be retrieved from surveys due to insufficient information(these participants are also not included in our study).
