# Supplementary material for: Effects of smoking on genome-wide DNA methylation profiles: A study of discordant and concordant monozygotic twin pairs
Source: eLife. 2023 Aug 10;12:e83286. doi: 10.7554/eLife.83286 (PMC10501767; doi:10.7554/eLife.83286)
Supplement: Source code 1. [file elife-83286-code1.zip › SourceCode1.docx]

**Analysis code EWAS in smoking discordant MZ twin pairs**

**######STEP 1 RESIDUALIZE METHYLATION DATA ############**

#==============================================================================================================================================================================================#

# * script uses 2 data objects: 1=methylation data. 2=Phenotype/covariate data.

# * methylation data is on the beta-value scale (0-1). Bad probes and bad samples have been removed. Data have been normalized.

# * methylation data contains numeric values. Missing values are denoted by NA.

# * methylation data has the following format: rows=samples, columns= methylation sites. The rownames are the sample identifiers.

# * methylation data has the following column names: Illumina probe ID (e.g. "cg1234567")

# * missing values in your phenotype and covariates are denoted by NA.

# * phenotype/covariate data has the following format: rows=individuals, columns=variables (covariates/phenotype). The rownames are the sample identifiers.

# * in code below, the methylation data object (1) is called "MethBetavalues" and is stored in Blood.450k.Betas.RData, the phenotype/covariate data object (2) is called "dat" and is stored in Covariates.RData

#==============================================================================================================================================================================================#

rm(list = ls(all = TRUE))

options(stringsAsFactors = FALSE)

# load data

load("Blood.450k.Betas.RData")

load("Covariates.RData")

#put objects in same order

dat <- dat[rownames(MethBetavalues),]

#extract necessary covariates

Sample_Plate <- as.factor(dat$Sample_Plate)

Array_rownum <- as.numeric(dat$Array_rownum)

Neut_Perc <- as.numeric(dat$Neut_Perc)

Mono_Perc <- as.numeric(dat$Mono_Perc)

Eos_Perc <- as.numeric(dat$Eos_Perc)

CpGi <- rep(NA,times=dim(dat)[1])

data <- data.frame(Sample_Plate,Array_rownumNeut_Perc,Mono_Perc,Eos_Perc, CpGi)

rownames(data) <- rownames(dat)

# select samples without missing data

select <- which(!is.na(data$Mono_Perc) & !is.na(data$Eos_Perc) & !is.na(data$Neut_Perc))

data <- data[select,])

# Linear model

LMJ <- function (data) { lm(CpGi ~ Mono_Perc+Eos_Perc+Neut_Perc+Array_rownum+Sample_Plate, na.action=na.omit,data=data)}

# check if objects are in the same order

table(rownames(data)==rownames(MethBetavalues))

#create output object “residuals”

Ncpgs<-ncol(MethBetavalues)

Ncpgs

Nsubjects <- nrow(data)

Nsubjects

residuals <- matrix(NA,Ncpgs,Nsubjects)

colnames(residuals) <- rownames(data)

rownames(residuals) <- colnames(MethBetavalues)

# Run linear model for all CpGs and save residuals

for (i in 1:Ncpgs)

{

data$CpGi <- MethBetavalues[rownames(data),i]

linearmodel <- LMJ(data)

for (k in 1:length(linearmodel$residuals))

{ residuals[i,names(linearmodel$residuals)[k]] <- linearmodel$residuals[k]

}

}

**######STEP 2 Paired T-test #########**

# script to compare DNA methylation within smoking discordant pairs (twin 1= never smoker, twin 2=current smoker)

# * script uses 2 data objects: 1= M_nonsmokingtwin=Methylation data residuals (residualized for covariates) of the non-smoking twin. 2= M_smokingtwin= Methylation data residuals (residualized for covariates) of the current smoking co-twins. Rows=samples, Columns=CpGs. Column names=CpG identifiers. The 2 objects are in the same order (that is, ordered by twin pairs, i.e. row 1=twin pair 1, row 2=twin pair 2 etc.).

NCpGs <- colnames(M_nonsmokingtwin)

# create object to store output

results <- matrix(nrow= NCpGs, ncol=5)

for (i in 1: NCpGs)

{

res <- t.test(M_nonsmokingtwin [,i], M_smokingtwin [,i], paired=T)

results[i,1] <- res$estimate

results[i,2] <- res$p.value

results[i,3] <- res$conf.int[1]

results[i,4] <- res$conf.int[2]

results[i,5] <- res$statistic

}

results <- data.frame(colnames(NCpGs),results)

colnames(results) <- c("cgid","MeanDifference","pvalue","95confint_L","95confint_H","statistic")
